# *In silico *panning for a non-competitive peptide inhibitor

**DOI:** 10.1186/1471-2105-8-11

**Published:** 2007-01-12

**Authors:** Yukiko Yagi, Kotaro Terada, Takahisa Noma, Kazunori Ikebukuro, Koji Sode

**Affiliations:** 1 Department of Biotechnology, Tokyo University of Agriculture and Technology, 2-24-13 Naka-machi, Koganei, Tokyo, Japan; 2 Product Development Dept, Medical & Biological Laboratories Co., 3-5-10 Marunouchi, Nakaku, Nagoya, Japan

## Abstract

**Background:**

Peptide ligands have tremendous therapeutic potential as efficacious drugs. Currently, more than 40 peptides are available in the market for a drug. However, since costly and time-consuming synthesis procedures represent a problem for high-throughput screening, novel procedures to reduce the time and labor involved in screening peptide ligands are required. We propose the novel approach of '*in silico *panning' which consists of a two-stage screening, involving affinity selection by docking simulation and evolution of the peptide ligand using genetic algorithms (GAs). *In silico *panning was successfully applied to the selection of peptide inhibitor for water-soluble quinoprotein glucose dehydrogenase (PQQGDH).

**Results:**

The evolution of peptide ligands for a target enzyme was achieved by combining a docking simulation with evolution of the peptide ligand using genetic algorithms (GAs), which mimic Darwinian evolution. Designation of the target area as next to the substrate-binding site of the enzyme in the docking simulation enabled the selection of a non-competitive inhibitor. In all, four rounds of selection were carried out on the computer; the distribution of the docking energy decreased gradually for each generation and improvements in the docking energy were observed over the four rounds of selection. One of the top three selected peptides with the lowest docking energy, 'SERG' showed an inhibitory effect with *K*_i _value of 20 μM. PQQGDH activity, in terms of the *V*_max _value, was 3-fold lower than that of the wild-type enzyme in the presence of this peptide. The mechanism of the SERG blockage of the enzyme was identified as non-competitive inhibition. We confirmed the specific binding of the peptide, and its equilibrium dissociation constant (*K*_D_) value was calculated as 60 μM by surface plasmon resonance (SPR) analysis.

**Conclusion:**

We demonstrate an effective methodology of *in silico *panning for the selection of a non-competitive peptide inhibitor from small virtual peptide library. This study is the first to demonstrate the usefulness of *in silico *evolution using experimental data. Our study highlights the usefulness of this strategy for structure-based screening of enzyme inhibitors.

## Background

According to market research, the potential of peptide therapeutics has recently intensified [1-3]. Worldwide, there are more than 40 marketed peptides, with about 270 peptides in clinical phase testing, and about 400 peptides in advanced preclinical phases [1]. Natural peptides, such as insulin, vancomycin, oxytocin, and cyclosporine, and synthetically produced peptides, such as Fuzeon (enfuvirtide) and Integrilin (eptifibatide), are among the approved peptide-based drugs. Compared to low-molecular-mass chemical drugs, peptide drugs offer several advantages, such as high specificity, minimization of drug-drug interactions, lower accumulation in tissues, lower toxicity, and biological diversity. However, peptides also have some disadvantages, which include low oral bioavailability, lower stability, higher risk of immunogenic effects, difficulties associated with delivery due to rapid clearance from the body, and costly synthesis. Recently, several novel and interesting approaches to deliver protein-based drugs through the skin have been reported [4]. Since peptides require costly synthesis, high-throughput screening (HTS) of numerous peptides from combinatorial libraries is inefficient. Therefore, novel procedures that require less effort for the screening of peptide ligands are required. From this point of view, structure-based computational drug design is an effective methodology. Recent advances in protein structure determination, achieved either through X-ray crystallography or NMR, are providing informative data related to the design of useful drugs based on these proteins. The identification of the binding sites on these newly determined protein structures have led to the development of a variety of docking strategies. There are numerous reports of drug discovery from small molecule ligand libraries [5,6], although it is difficult to calculate the docking energies of all the peptide sequence patterns, as they show enormous diversity. Therefore, we focused on the use the genetic algorithms (GAs) to reduce the redundancy of the selection procedure.

GAs represent a class of algorithms that mimic some of the major characteristics of Darwinian evolution [7,8]. GAs are based on the process of genetic evolution observed in biological systems, in which three successive operations, selection, crossover, and mutation, are performed on a set of strings. GAs provide an effective means of exploring the conformational space of flexible molecules. GAs also provide an effective approach to protein folding [9], identification of the biomolecular conformation space [10], docking methodology [11,12], optimization of lead compounds [8,13], chemical evolution of combinatorial chemistry [14], and identification of receptor-ligand binding sites [15].

**Figure 1:**
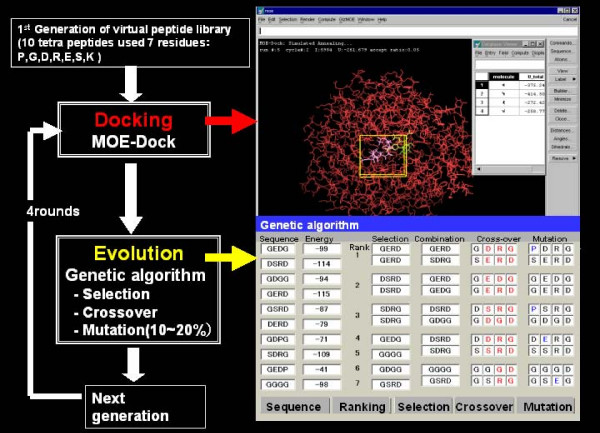
**A schematic diagram of the *in silico *peptide evolution system**. The docking calculation program and genetic algorithms (GAs) were combined to evolve the peptide ligand on the computer. For the docking program, we used the MOE-Dock software, which is based on the simulated annealing method. GAs were used to evolve the peptide to produce the next generation. Four rounds of peptide evolution were performed in this study.

We have previously reported the application of GAs to select a peptide inhibitor [16], an α-helix-forming peptide [17], and a DNA aptamer with higher-order structure [18-20]. From the result of those studies, it is clear that GAs are useful for the efficient selection of molecules that have a desired property or function, since we can reduce the number of rounds of evaluation. In the present study, we have focused on the application of GAs for effective peptide ligand selection from a docking simulation. Belda et al. [21] have also reported a combination of computational docking and combinatorial experimental screening but have not provided experimental data. We propose an effective approach to derive peptide ligands, which we call '*in silico *panning'. By combining the docking study and GAs, we are able to identify promising peptide ligands from a small virtual peptide library with less effort (Figure 1).

To demonstrate *in silico *panning, we chose the water-soluble quinoprotein glucose dehydrogenase (PQQGDH) from *Acinetobacter calcoaceticus *as the target protein. Mainly due to its high catalytic activity and non-dependence on oxygen as an electron acceptor, PQQGDH has replace glucose oxidase (GOD) as the major enzyme used in glucose sensor systems [22]. The only aspects in which PQQGDH is inferior to GOD are substrate specificity and operational stability. PQQGDH shows high activity not only for glucose, but also for disaccharides, such as lactose and maltose, as substrates. Improvements in substrate specificity are expected to lead to the development of more-sensitive glucose sensors. Therefore, PQQGDH engineering has been carried out in our research group [22,23]. We have already reported mutants of PQQGDH (Glu277Lys, Asn452Thr, Asp167Glu, and Asp167Glu/Asn452Thr) that show improved substrate specificities [24,25].

**Figure 2:**
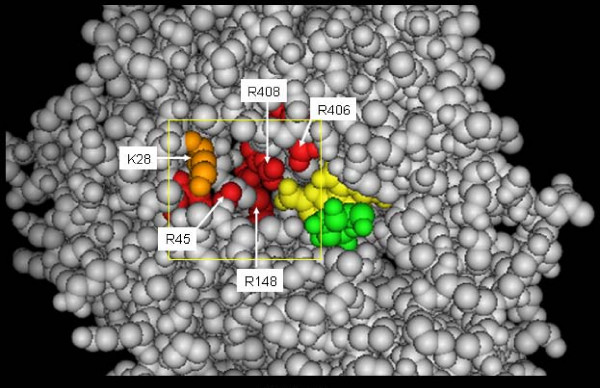
**Defining a docking box next to the glucose-binding site**. The 3D structure of water-soluble quinoprotein glucose dehydrogenase from *Acinetobacter calcoaceticus *(PQQGDH: PDB entry code 1CQ1) is shown in gray. PQQ and glucose are represented in yellow and green, respectively. The large pocket next to the glucose-binding site of PQQGDH comprises Arg148, Arg406, Arg408, Arg45 (red) and Lys28 (orange). The docking box was set as the target area for this pocket. The docking box is shown as a yellow rectangular solid. The size of the docking box was fixed as a 37 Å

We have also been proposing quaternary structure engineering of proteins, which is the control of protein function using an artificial subunit. In nature, several enzymes are composed of subunits that form an active quaternary structure, which endows a higher order of function. The strategy of quaternary structure engineering is to mimic the native enzyme using a peptide ligand as an artificial subunit with a novel function. From a phage display peptide library, we previously identified a peptide with seven amino acids (Thr-Thr-Ala-Thr-Glu-Tyr-Ser) that narrowed the substrate specificity of PQQGDH without significant loss of enzyme activity [26]. In the present study, *in silico *peptide evolution was performed to investigate a new methodology for protein modification. Our ultimate objective is the selection of a non-competitive peptide ligand that does not interrupt glucose binding but inhibits the interaction between disaccharides, such as maltose and lactose. The disaccharides appear to access a large pocket structure next to the catalytic site of PQQGDH, which comprises Arg148, Arg406, Arg408, Arg45, and Lys28 (Figure 2). We defined this large pocket as the docking field. From size of this pocket, tetra peptide was designed to be the most suitable size as initial tetra peptide library. Then, the peptide ligand was selected through a combination of the docking simulation and GAs on the basis of binding indicators.

We chose the Molecular Operating Environment of docking (MOE-Dock) system with simulated annealing method as a starter system [27,28]. Simulated annealing is a global optimization technique that is based of the Monte Carlo method. It explores various states of a configuration space by generating small random changes in the current state and then accepting or rejecting each new state according to the Metropolis criterion [29].

## Results

### Docking energy transition in combination with *in silico *evolution

**Figure 3:**
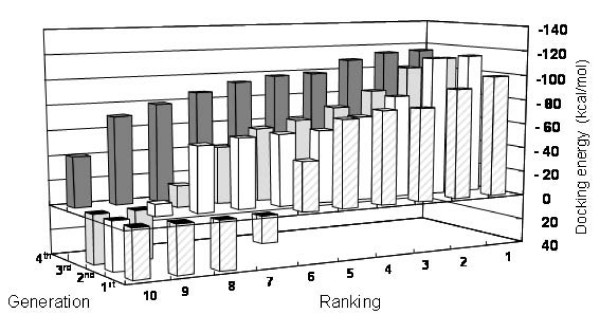
**The distribution of docking energies in each generation**. The ranks of the peptide ligands in each generation are shown on the X- and Y-axes. The Z-axis shows the docking energies, which were calculated as follows: docking total energy = electrostatic energies + Van der Waals energies + the energy of the (flexible) ligand. Low docking energy means high binding ability of the ligand.

One of the key steps in docking is how to define the user-specified three-dimensional docking box (3D docking box). To select the non-competitive peptide ligand, we designated the large pocket next to the glucose-binding site (Figure 2). Then the peptide ligand was selected by *in silico *panning; a combination of the docking simulation and GAs on the basis of binding indicators. In all, four rounds of selection were carried out from the beginning of ten virtual peptide library on the computer. The docking energy (fitness) results for each selection round are listed in Table 1. In MOE-Dock, electrostatic energies and Van der Waals energies between the target and ligand are calculated by simulated annealing. The docking energy values were calculated as the sum of the electrostatic, Van der Waals energies and the flexibility of the ligand itself. Low docking energy indicates high binding ability. The distributions of the docking energies in each generation are illustrated in Figure 3; each docking energy decreased gradually as peptide evolution progressed. After 4^th ^round of peptide evolution, the docking energies of most of the peptides were lower than those in the 1^st^–3^rd ^round. Peptide evolution and the direction of the selection proceeded successfully using the GA.

**Table 1:** The docking energy of peptides in each generation

1round	Docking energy (kcal/mol)		2round	Docking energy (kcal/mol)		3round	Docking energy (kcal/mol)		4round	Docking energy (kcal/mol	
No.1	GDGD	-99	No.1	SERG	-114	No.1	GDRD	-110	No.1	GERD	-115
No.2	GEPR	-90	No.2	GDPR	-113	No.2	GDRG	-103	No.2	DSRD	-114
No.3	PSRG	-76	No.3	GEGD	-83	No.3	GEGG	-85	No.3	SDRG	-109
No.4	DGDG	-76	No.4	RGDG	-68	No.4	RGDD	-73	No.4	GEDG	-99
No.5	DDGR	-71	No.5	DDRG	-59	No.5	DEGD	-64	No.5	GGGG	-98
No.6	SDEE	-40	No.6	DDDR	-58	No.6	DDDG	-59	No.6	GDGG	-94
No.7	DDDD	22	No.7	DDGD	-57	No.7	DDPR	-46	No.7	GSRD	-87
No.8	RDKP	52	No.8	PSGR	-53	No.8	KEPR	-17	No.8	DEGG	-79
No.9	ERKS	88	No.9	PSEE	-10	No.9	GKRR	785	No.9	GDPG	-71
No.10	SREK	529	No.10	EERD	160	No.10	SERR	50397	No.10	GEDP	-41

Each peptide sequence and docking energy of 1–4^th ^generation was summarized.

### Homologies of highly ranked peptide ligands from the docking simulation

**Figure 4:**
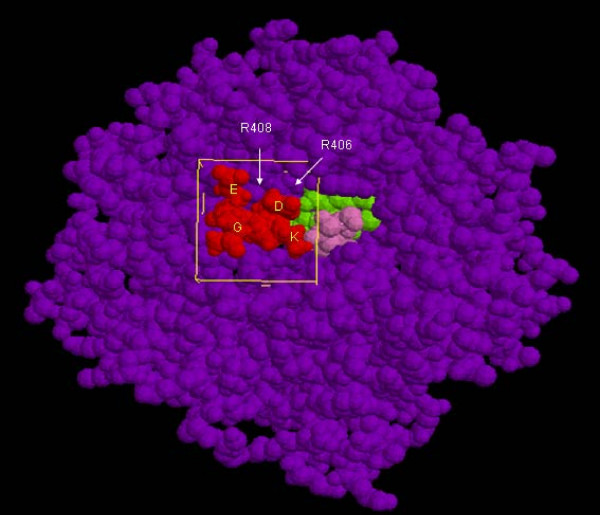
**Peptide GEKD docked in PQQGDH**. GDH, PQQ, and glucose are represented in purple, green, and pink, respectively. The GEKD peptide is displayed in red. The docking box is shown as a yellow rectangular solid.

In comparisons of the homologies of high-scoring peptides, the sequences appeared to converge (Table 2). The peptide homologies converged almost completely after the 4^th ^round of peptide evolution. A negatively charged residue was revealed at the third position with a probability of 80%. Furthermore, 60% of the peptides contained positively charged residues at the fourth position. On the other hand, lysine residues did not appear at the fourth position during the four rounds of selection due to deflection of evolution. For this reason, we designed additional peptides, and re-calculated their docking energies. Finally, the top three peptides and negative controls were synthesized for further analysis. Figure 4 displays an image of PQQGDH interacting with peptide GEKD, which was derived by MOE-Dock.

**Table 2:** Homologies of selected peptide sequences

4^th^round peptides			Additional round peptides			Synthesized peptides		
Rank	Peptide sequence	Docking energy (kcal/mol)	Rank	Peptide sequence	Docking energy (kcal/mol)	Rank	Peptide sequence	Docking energy (kcal/mol)
1	GERD	-115	1	GEKD	-149	1	GEKD	-149
2	SERG	-114	2	SEKD	- 88	2	GERD	-115
3	DSRD	-114	3	GDKD	- 87	3	SERG	-114
4	GDPR	-113	4	DSKD	- 75	38	DDDD	22
5	GDRD	-110	5	GEKE	- 22			
6	SDRG	-109						
7	GDRG	-103						
8	GDGD	-99						
9	GEDG	-99						
10	GGGG	-98						

The homologies of selected peptides ranked in the top 10 after the 4^th ^round of evolution were listed to the left. Additional peptides that contained a lysine (K) residue at the third position were also listed on the middle. The final rank and the synthetic peptide sequences were shown on the right. The DDDD peptide was used as a negative control.

### Effects of synthetic peptide ligands on PQQGDH activity

**Figure 5:**
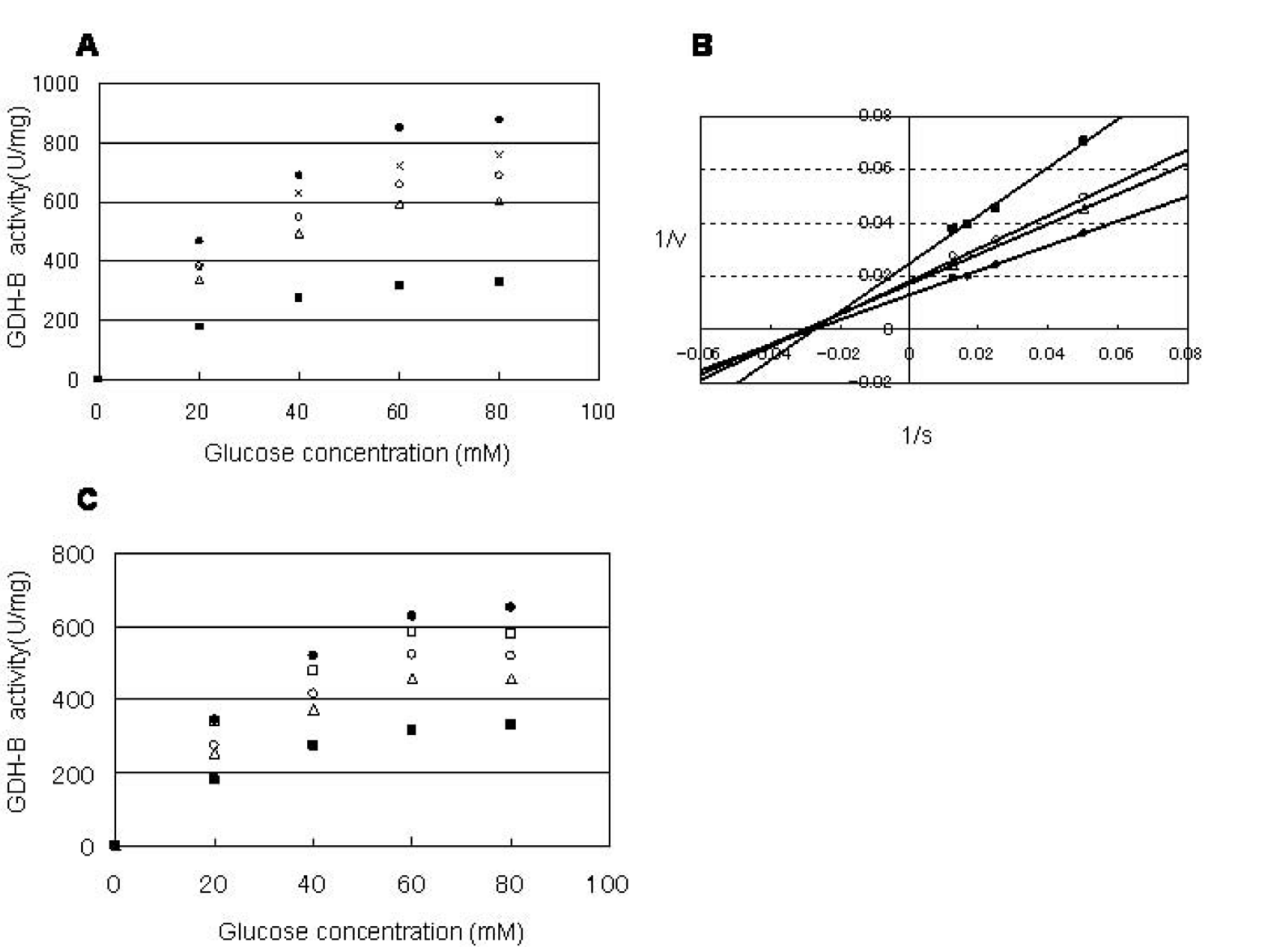
**PQQGDH activity for glucose in the presence of individual peptides**. (A) The PQQGDH activities were measured in the presence of the synthetic peptides. The enzymatic activities for glucose are shown in the SV plot. The enzyme assay was performed with 0.57 nM PQQGDH and 20 μM of each peptide. The samples contained the following: no peptide, (●); GEKD, (○); GERD, (□); SERG, (■); DDDD, (×). (B) Linewaver-Burk plot of PQQGDH activities in the presence of 0 μM SERG (●), 2 μM SERG (△), 10 μM SERG (○), and 20 μM SERG (■). All of the correlation coefficients (R^2^) were > 0.98. The X-axis shows the reciprocal values of the glucose concentration, and the Y-axis indicates the reciprocal values of the kinase activity. (C) PQQGDH activities of the wild-type (●), with 0.2 μM peptide (×), with 1 μM peptide (□), with 2 μM peptide (△), with 20 μM peptide (■) were plotted against different glucose concentrations. The SERG peptide was used in the concentration range of 0.2–20 μM.

As shown in Figure 5A, 20 μM of peptide SERG showed the strongest inhibitory effect on the target enzyme. The same concentration of GEKD or GERD also showed higher inhibition than the control peptide DDDD. The velocity at maximal concentrations of substrate(*V*_max_) value of SERG (473 U/mg) was 3-fold lower then that of the wild-type enzyme (1272 U/mg), while the affinity of the enzyme for the substrate (*K*_m_) values were the same (wild-type, 33.2 mM; SERG, 31.2 mM). GEKD and GERD gave only slight inhibition of the target, with *V*_max _values of 962 U/mg and 849 U/mg, respectively. Based on the results of the Lineweaver-Burk plot (Figure 5B), SERG was deemed to show non-competitive inhibition of the substrate. Non-competitive inhibitors do not affect the combination of the substrate with the enzyme, but it does affect the velocity. In a *pure *non-competitive system, the substrate has an identical affinity for both the Enzyme(E)-Inhibitor(I) complex and enzyme. Unlike the Enzyme(E)-Substrate(S) complex, the E-I-S complex cannot convert the substrate to product. Therefore, the *K*_m _value is unchanged while *V*_max _is lowered. The data show that SERG decreases the rate constant for product formation, while *K*_m _shows a constant value. It follows that SERG is not a glucose competitor. On the other hand, GERD and GEKD showed 'mixed-type inhibition' (data not shown). The peptide SERG showed dose-dependent inhibition (Figure 5C) and its enzyme inhibition constant(*K*i) value was calculated as 20 μM from the Dixon plot (Table 3). All of the *K*i values were calculated using the KaleidaGraph software (Synergy Software, Boston, MA, USA). Although the selected peptides showed potent inhibition, they did not show significantly decreased substrate specificities for disaccharides (data not shown).

**Table 3:** Inhibition and binding constants of peptides

Peptide sequence	Docking energy (kcal/mol)	*K*_*i *_I(nhibition constant)	*K*_*D *_(Binding constant)
GEKD	-149	50 *μ*M	155 *μ*M
GERD	-115	40 *μ*M	-
SERG	-114	20 *μ*M	60 *μ*M
DDDD	22	> 100 *μ*M	-

The *K*i values were derived from the Dixon plot. The *K*_D _values were determined from the Scatchard plot.

### Binding parameters of selected peptides by surface plasmon resonance (SPR)

During the SPR experiments, the binding assay for the biotinylated peptides (GEKD and SERG) and PQQGDH was processed by the BIAcoreX instrument (BIAcore AB, Uppsala, Sweden). The equilibrium dissociation constant (*K*_D_) value used to evaluate the enzyme-peptide binding affinity was determined by Scatchard plot (Table 3). For each trial, the signal was corrected for the control surface response. We were able to confirm peptide binding to the enzyme, and the *K*_D _values of peptides GEKD and SERG were calculated as 155 μM and 60 μM, respectively.

## Discussion

In this paper, we introduce the idea of *in silico *panning, which is a novel strategy to select peptide inhibitors by combining a docking simulation and GA. Setting the docking field next to the substrate binding site, we were able to obtain a non-competitive peptide inhibitor from a small virtual peptide library. In this study, the non-competitive peptide inhibitor was successfully and with less effort selected from a small virtual peptide library using *in silico *panning. This methodology can be used to screen an allosteric binding peptide inhibitor at an early stage.

Information derived from X-ray crystallography, NMR spectroscopy, and homology modelling greatly facilitates the rational design of selective and potent inhibitors. In addition, rapid identification of lead molecules and optimization of inhibitors are facilitated by large combinatorial libraries and high-throughput screening. However, successful virtual screening of chemical libraries in the drug discovery process requires a sufficiently large and chemically diverse library of compounds. In addition, the selection of promising peptide inhibitors from such large libraries involves significant cost and effort [1]. Therefore, we focused on the use of the GAs to reduce redundancy during the selection procedure.

In *in silico *panning, all of the selection processes are carried out by the computer. First, we calculate the affinity of the peptide ligand from docking simulation, and then evolve the sequence by GA, which mimic Darwinian evolution. In the present study, the initial group involved ten virtual tetrapeptides designed randomly from seven different amino acid residues (Arg, Lys, Asp, Glu, Ser, Pro, and Gly). The Arg, Lys, Asp, Glu, and Ser residues were mainly chosen to form the electrostatic and hydrogen interaction. In addition, hydrophobic Pro residues were appeared frequently in a phage display peptide library in a previous study [26], so we chose this residue. Gly was chosen to increase the flexibility of the ligand. The large pocket next to the glucose binding site of PQQGDH is mainly composed of hydrophilic residues, including Arg148, Arg406, Arg408, Arg45, and Lys28. In this study, to simplify the experimental method and concentrate on the hydrophilic interactions, we chose mainly hydrophilic residues for designing the initial peptide library. In the near future, a more complex library, containing several hydrophobic residues, will be used. Totally, four rounds of selection were carried out, and 45 types of tetrapeptide encompassing seven different amino acid residues were evolved by the computer. Since this covered only 18.7% of the 2401 possible tetrapeptide combinations, we conclude that the peptide ligands were efficiently selected from this small library by *in silico *panning. This is the first study to demonstrate the usefulness of *in silico *evolution using experimental data.

We have reported previously the identification from a phage display peptide library of a 7-mer peptide that causes PQQGDH substrate specificity towards disaccharides to decrease significantly [26]. We expected that some of the evolved tetrapeptides would improve the substrate specificity of PQQGDH. Although we were not able to obtain a peptide that improved substrate specificity, we obtained a non-competitive peptide inhibitor of the target protein. The selected peptide inhibits the enzyme activity not only for glucose, but also for disaccharides (data not shown). Thus, the substrate specificity does not change in the presence of the peptide ligand. To overcome these problems, we have to select peptide ligands that bind to the target pocket without inhibiting glucose binding. It is possible to calculate the binding of glucose or disaccharides to target area after derivation of a candidate peptide using the docking simulation. Thus, we may be able to choose a ligand that decreases only the reactivities for disaccharides.

In this study, we present a valuable method for the selection of a non-competitive peptide inhibitor. For target enzyme that are highly homologous, particularly at the catalytic site, target-specific inhibition is possible through the use of an allosteric or non-competitive inhibitor, which does not bind to the catalytic site but has inhibitory activity. Thus, a non-competitive inhibitor sometimes became a specific inhibitor for protein having high homogeny [30]. Although an RNA aptamer and peptide inhibitor with non-competitive and specific inhibitory activities have been selected from a pooled random sequence library [30,31], a method that allows one to choose a non-competitive inhibitor *de novo *is more efficient. In our study, by setting the enzyme pocket, which appears to be an allosteric binding site, as the docking area, the non-competitive peptide inhibitor was obtained as expected. The top three selected peptides that were evolved by binding index in the docking study showed much greater inhibition compared to the negative control, DDDD. The most effective peptide, SERG showed potent inhibition with a *K*_i _value of 20 μM and *K*_D _value of 60 μM. In MOE-Dock, the docking energies were calculated as the sum of three energies, electrostatic energies, van der Waals energies and energy of the (flexible) ligand. Analysis of the individual components of the calculated binding energies (Table 4) shows that the top three peptides have lower electrostatic energies and energies of flexibility than the negative control peptide DDDD. All of the ten peptides that were expected to have high affinity also showed low electrostatic energies and energy of flexibility (data not shown). We initially expected that the peptide DDDD might show high affinity since the pocket next to the glucose-binding site in PQQGDH contains five positively charged residues. However, the docking energy of DDDD was ranked 38^th ^in the docking study (Table 1). The peptide DDDD showed weak electrostatic interaction with PQQGDH due to the low flexibility that comes from the intramolecular ionic repulsion (Table 4). In fact, the peptide DDDD did not show remarkable inhibitory effect, suggesting that flexibility of a peptide inhibitor may play an important role in the interaction with the target molecule.

**Table 4:** The docking energy detail of peptides

Peptide sequence	U-ele (kcal/mol)	U-vdw (kcal/mol)	U-int (kcal/mol)	Docking energy (kcal/mol)
GEKD	- 76	- 10	- 61	-149
GERD	- 56	- 10	- 50	-115
SERG	- 62	- 9	- 44	-114
DDDD	34	- 16	3	22

U-ele = Electrostatic energies between target and ligand

U-vdw = Van der Waals energies

U-int = energy of the (flexible) ligand

The docking energy was calculated for the sum of three energy, electrostatic energies, van der Waals energies and energy of the (flexible) ligand. Each energy listed in the table was rounded off to the whole number.

In future studies, the use of longer sequences that form higher-order structures will generate more specific peptide inhibitors from *in silico *panning. This method has strong potential to become a useful tool for structure-based non-competitive inhibitor screening.

## Conclusion

We have demonstrated the potential of *in silico *panning for selecting non-competitive peptide inhibitors in a more efficient manner. By choosing a target region next to catalytic site in the docking study and then evolving the peptide ligand on the basis of binding indicators, we succeeded in obtaining a non-competitive peptide inhibitor. The most effective peptide showed potent inhibition with a *K*_i _value of 20 μM and *K*_D _value of 60 μM. Our *in silico *panning approach should become a useful tool for screening structure-based enzyme inhibitors. This methodology has excellent potential for the screening of non-competitive peptide ligands for allosteric binding sites of an enzyme in the early stages.

## Methods

### Chemicals and reagents

Glucose, phenazine methosulfate (PMS), 2,6-dichlorophenolindophenol (DCIP), EDTA, and calcium chlorite were obtained from Kanto Kagaku (Tokyo, Japan), 3-(N-morpholino) propane sulfonate (MOPS) was from Dojin (Kumamoto, Japan), and pyrroloquinoline quinone was from Mitsubishi Gas Chemical Company (Tokyo, Japan). The sensor chip SA was from BIAcore AB (Uppsala, Sweden). All other regents were of analytical grade. The Molecular Operating Environment (MOE) was obtained from the Chemical Computing Group (Quebec, Canada).

### Design of the initial peptide library

Ten tetrapeptides encompassing seven different amino acid residues were identified in the first generation. The large pocket next to the catalytic site of PQQGDH, which is mainly composed of the conserved residues of Arg148, Arg406, Arg408, Arg45, and Lys28, was designated as the docking field. Arg, Lys, Asp, Glu, and Ser residues were chosen to form electrostatic and hydrogen interactions with the residues in the large pocket. The hydrophobic amino acid proline appeared frequently in the phage display peptide library examined in a previous study [26], so we also chose this residue. Gly was added to increase the flexibility of the ligand.

### Calculation of docking energies of peptide ligands for the enzyme catalytic site

The Molecular Operating Environment of docking (MOE-Dock; [27,28]) was used to calculate the docking energies between peptide ligands and enzyme catalytic site. MOE-Dock is used to search for favorable binding configurations between a small ligand and a macromolecular target. The peptides were drawn by the MOE software and stored in the database.

The PDB structure of GDH (PDB entry code 1CQ1) and stored peptide were imported at the start of the docking program. Since not all X-ray crystallographic files contain hydrogen atoms, we added them to the protein using the MOE modelling suite before carrying out the docking studies. Minimizing contacts for hydrogen, the structures were subjected to an AMBER94 energy minimization protocol. The docking energy calculation was carried out within a user-specified three-dimensional docking box (3D docking box) using the simulated annealing method under the MMFF94 force field. The energy grids for docking were generated as *grid-based potential fields *by the MOE-Dock program, to reduce the calculation time.

We selected the large pocket next to the catalytic site of PQQGDH, which includes Arg148, Arg406, Arg408, Arg45, and Lys28, as the target area in the 3D docking box. After importing the peptide to the 3D docking box, we initiated the calculation of docking energies. Each docking energy value was calculated as the sum value of the electrostatic, Van der Waals, and flexibility energies. The interaction energy was calculated using the electrostatic and Van der Waals potential fields sampled on a grid overlaying the 3D docking box. The 3D docking box was interpolated at the atom positions by tri-linear interpolation. The Van der Waals parameters were taken from the currently active force field. The electrostatic field was calculated in the Coulombic manner using the current dielectric. MOE-Dock performed 25 independent docking runs, and wrote the resulting conformations and their energies to a molecular database file. The lowest docking energy conformation for each peptide was chosen for GA evolution. All of the docking simulations were carried out on a personal computer.

### Evolution of peptide ligands using GA

After calculation of the docking energies of the initial ten peptides (described in the *Methods *section), the peptides were evolved by GA, to produce the next generation. First, each of the ten peptides was ranked according to its calculated docking energy, and then the top seven peptides were remained for evolution by GA. The GA program then duplicated the top three sequences and formed a pair with the remaining (4^th^–7^th^) peptides. Then ten peptides were recombined into the sequence. Finally, a 10–20% mutation factor was introduced to increase sequence variety in the next generation. Each process from docking to GA evolution was repeated for four rounds.

### Characterization of peptide effects on PQQGDH activity

The three peptides with the lowest docking energies, GEKD, GERD, and SERG, and the control peptide DDDD were synthesized by Qiagen (Chatsworth, CA, USA). The PDB file of PQQGDH is a monomer and does not contain Ca^2+^. In general, purified PQQGDH is a mixture of an apo- and holo-type enzyme. Therefore, we dialyzed the enzyme against 100 mM EDTA and 10 mM MOPS-NaOH buffer for 2 days, to remove the Ca^2+ ^ions. Each synthesized peptide was prepared at concentrations of 0.2, 1, 2, 10, and 20 μM. The prepared peptides and 0.57 nM of apo-PQQGDH were incubated in 10 mM MOPS-NaOH buffer (pH 7.0) for 10 min at room temperature. PQQGDH activity was measured using 0.6 mM PMS and 0.06 mM DCIP. Decreased in the absorbance of DCIP at 600 nm at various concentrations of glucose were monitored, and the kinetic parameters of PQQGDH were as described previously [26]. The kinetic parameters and *K*i values were calculated using the KaleidaGraph software.

### Surface plasmon resonance (SPR) data analysis

The GEKD and SERG peptides, which showed inhibitory effects on GDH, were synthesized with a biotin modification (Qiagen).

The binding features of the peptide ligand to PQQGDH have been studied using SPR technology based on the BIAcoreX instrument (BIAcore). The biotinylated GEKD and SERG peptides were immobilized onto the Biacore SA sensor chip using the streptavidin-biotin interaction, and 120 μg/ml of each peptide in 100 mM NaCl, 10 mM phosphate buffer (pH 7.0) was applied a flow rate of 5 μl/min for 16 min. The resonance signals of peptides GEKD and SERG reached 273 and 300 resonance units (RUs). PQQGDH for use as an analyte was prepared at concentrations of 446 μg/ml, 223 μg/ml, 89 μg/ml, and 47 μg/ml in 10 mM MOPS-NaOH buffer (pH 7.0). Analytes were injected into the immobilized peptides at a flow rate of 20 μl/ml for 4 min at 25°C. All of the SPR experiments were performed in 10 mM MOPS-NaOH buffer (pH 7.0), and 0.5% SDS was chosen for surface regeneration. The sensor-gram signal of PQQGDH with each peptide was normalized by the nothing-immobilized sensor chip. The equilibrium dissociation constant (*K*_D_) value was calculated from the Scatchard plot.

## Abbreviations

PQQGDH, water-soluble quinoprotein glucose dehydrogenase from *Acinetobacter calcoaceticus*; PQQ, pyrroloquinoline quinine; GAs, genetic algorithms; NMR, nuclear magnetic resonance; MOE-Dock, Molecular Operating Environment of docking; *K*_D, _equilibrium dissociation constant; *K*i, enzyme inhibition constant; SPR, surface plasmon resonance.

## Authors' contributions

YY carried out the docking study and determined the peptide inhibitory activity for PQQGDH and drafted the manuscript. KT carried out the SPR study. TN developed the GA program. KI and KS conceived the study and participated in its design and co-ordination. All of the authors have read and approved the final manuscript.

## References

[B1] V M (2005). WATCHING PEPTIDE DRUGS GROW UP. CHEMICAL& Engineering News.

[B2] Latham PW (1999). Therapeutic peptides revisited. Nat Biotechnol.

[B3] Loffet A (2002). Peptides as drugs: is there a market?. J Pept Sci.

[B4] Y C, Y S, X G, C Z, W Y, M M, S L, M Z, LP W (2006). Transdermal protein delivery by a coadministered peptide identified via phage display.. Nat Biotechnol.

[B5] Scapin G (2006). Structural biology and drug discovery. Curr Pharm Des.

[B6] Hou T, Xu X (2004). Recent development and application of virtual screening in drug discovery: an overview. Curr Pharm Des.

[B7] P W (1995). Genetic algorithms in molecular recognition and design. TIBTECH DECEMBER.

[B8] L W, S W, C B, K G (1995). Optimization of the Biological Activity of Combinatorial Compound Libraries by a Genetic Algorithm. Angew Chem Int Ed Engl.

[B9] Dandekar T, Argos P (1994). Folding the main chain of small proteins with the genetic algorithm. J Mol Biol.

[B10] AY J, FY L, DF W (1999). Three variations of genetic algorithm for searching biomolecular conformation space: Comparison of GAP 1.0, 2.0, and 3.0. JOURNAL OF COMPUTATIONAL CHEMISTRY.

[B11] Gardiner EJ, Willett P, Artymiuk PJ (2001). Protein docking using a genetic algorithm. Proteins.

[B12] Jones G, Willett P, Glen RC, Leach AR, Taylor R (1997). Development and validation of a genetic algorithm for flexible docking. J Mol Biol.

[B13] J S, M A, E J, M A, D W, J S, S C, A T (1996). Application of Gentetic Algorithms to Combinatorial Synthesis: A Computational Approach to Lead Identification and Lead Optimization. J Am Chem Soc.

[B14] AV E, MI N (1998). Use of molecular recognition to drive chemical evolution, Part 2. 
Mechanisms of an automated genetic algorithm implementation. CHEMISTRY-A EUROPEAN JOURNAL.

[B15] Shah PK, Buslje CM, Sowdhamini R (2001). Structural determinants of binding and specificity in transforming growth factor-receptor interactions. Proteins.

[B16] Yokobayashi Y, Ikebukuro K, McNiven S, Karube I (1996). Directed evolution of trypsin inhibiting peptides using a genetic algorithm. J Chem Soc ,Perkin Trans.

[B17] Zhang W, Loughran MG, Kanna S, Yano K, Ikebukuro K, Yokobayashi Y, Kuroda R, Karube I (2003). Exploration of structural features of monomeric helical peptides designed with a genetic algorithm. Proteins.

[B18] Noma T, Ikebukuro K, Sode K, Ohkubo T, Sakasegawa Y, Hachiya N, Kaneko K (2006). A screening method for DNA aptamers that bind to a specific, unidentified protein in tissue samples. Biotechnol Lett.

[B19] Noma T, Ikebukuro K (2006). Aptamer selection based on inhibitory activity using an evolution-mimicking algorithm. Biochem Biophys Res Commun.

[B20] Ikebukuro K, Noma T (2003). Screening of DNA aptamers inhibiting Taq DNA polymerase using algorithm mimicking evolution. Nucleic Acids Res Suppl.

[B21] Belda I, Madurga S, Llora X, Martinell M, Tarrago T, Piqueras MG, Nicolas E, Giralt E (2005). ENPDA: an evolutionary structure-based de novo peptide design algorithm. J Comput Aided Mol Des.

[B22] Igarashi S, Okuda J, Ikebukuro K, Sode K (2004). Molecular engineering of PQQGDH and its applications. Arch Biochem Biophys.

[B23] S. T, Igarashi S, F S, K* S (2005). Increasing stability of water-soluble PQQ glucose dehydrogenase by increasing hydrophobic interaction at dimeric interface. BMC Biochemistry.

[B24] Igarashi S, Hirokawa T, Sode K (2004). Engineering PQQ glucose dehydrogenase with improved substrate specificity. Site-directed mutagenesis studies on the active center of PQQ glucose dehydrogenase. Biomol Eng.

[B25] Igarashi S, Ohtera T, Yoshida H, Witarto AB, Sode K (1999). Construction and characterization of mutant water-soluble PQQ glucose dehydrogenases with altered K(m) values--site-directed mutagenesis studies on the putative active site. Biochem Biophys Res Commun.

[B26] Yoshida H, Yagi Y, Ikebukuro K, Sode K (2003). Improved substrate specificity of water-soluble pyrroloquinoline quinone glucose dehydrogenase by a peptide ligand. Biotechnol Lett.

[B27] Chang DT, Oyang YJ, Lin JH (2005). MEDock: a web server for efficient prediction of ligand binding sites based on a novel optimization algorithm. Nucleic Acids Res.

[B28] title W (2006). http://www.chemcomp.com/software-sbd.htm. http://www chemcomp com/software-sbd htm.

[B29] Metropolis N, Rosenbluth AW, Rosenbluth MN, Teller AH (1953). Equation of state calculations by fast computing machines.. Journal of Chem Phys.

[B30] Huang L, Sexton DJ, Skogerson K, Devlin M, Smith R, Sanyal I, Parry T, Kent R, Enright J, Wu QL, Conley G, DeOliveira D, Morganelli L, Ducar M, Wescott CR, Ladner RC (2003). Novel peptide inhibitors of angiotensin-converting enzyme 2. J Biol Chem.

[B31] Gal SW, Amontov S, Urvil PT, Vishnuvardhan D, Nishikawa F, Kumar PK, Nishikawa S (1998). Selection of a RNA aptamer that binds to human activated protein C and inhibits its protease function. Eur J Biochem.

